# Morphological and Allometric Changes in *Anguilla japonica* Larvae

**DOI:** 10.3390/biology11030407

**Published:** 2022-03-06

**Authors:** Min-Gyu Shin, Yong-Woon Ryu, Youn-Hee Choi, Shin-Kwon Kim

**Affiliations:** 1 Aquaculture Research Division, National Institute of Fisheries Science, 216, Gijanghaean-ro, Gijang-eup, Gijang-gun, Busan 46083, Korea; mingyushin7@gmail.com (M.-G.S.); ryuyw@korea.kr (Y.-W.R.); 2 Department of Fisheries Biology, Pukyong National University, Busan 48513, Korea; 3 Department of Marine Bio-Materials & Aquaculture, Pukyong National University, Busan 48513, Korea

**Keywords:** ontogenetic development, leptocephalus, morphology, allometry, marine larviculture

## Abstract

**Simple Summary:**

The freshwater eel *Anguilla japonica* is a commercially important in Northeast Asia. However, eels farming is entirely dependent on natural catches, and eel resources are declining. This study was conducted to provide baseline information on the development of mass seed production technology necessary for the conservation of species and the maintenance of aquaculture. This study was conducted for 200 days after hatching (DAH) and analyzed morphometry and allometry. In this study, cultured eel larvae stages were divided in size similar to wild eel larvae, but cultured eel differed from wild eel in growth rate and the number of preanal myomeres. In addition, as eel larvae rarely have mixed feeding periods, it is important to determine the optimal first feeding time. The eel larvae may need a change in diet type to prevent lower jaw deformity in the leptocephalus stage. As eel larvae changed to a willow leaf-like form, the relative growth pattern of the eel larval stages was unique. This growth pattern may reflect the early life history of long distance and diel vertical migration. Meanwhile, the inflection point in the body parts’ growth patterns showed only before 30 DAH and was similar to the period of mass mortality. Therefore, future studies should focus on developing an optimal feeding and rearing protocol from the first feeding to 30 DAH.

**Abstract:**

The freshwater eel *Anguilla japonica* is rapidly decreasing in number and has not yet been successfully mass produced. This may be at least partially attributable to the unique and long early life history of the eel. Therefore, we investigated its ontogeny of morphometry and growth pattern in larval stages to provide baseline information for understanding the early life history and improving seed rearing technology. This study was conducted for 200 days after hatching (DAH) and analyzed morphometry and allometry for eel larvae. The following cultured eel larval stages were identified: the yolk sac larvae stage (0–6 DAH, 3.23–6.85 mm total length (TL)), the pre-leptocephalus stage (7–30 DAH, 6.85–15.31 mm TL), and the leptocephalus stage (50–200 DAH, 15.31–60.06 mm TL). Cultured and wild eel larvae could be divided into characteristic larval stages at similar sizes. However, compared to wild eels, cultured eels had a slower growth rate and fewer preanal myomeres. Meanwhile, cultured eel larvae rarely had a mixed feeding period as the absorption of endogenous reserves was completed by 7 DAH. The lower jaw of eel larvae was significantly longer than the upper jaw from 50 DAH. In the pre-leptocephalus and leptocephalus stages, eel larvae showed continuous positive allometric growth at trunk height and tail muscle height with change to the willow leaf-like form. These growth characteristics may be the result of adaptation to the migration over long distances and to a diel vertical migration. The inflection point in the body parts growth patterns showed only before 30 DAH, and mass mortality appeared at this period. Therefore, to improve the growth and survival rates of cultured eel seed, it is necessary to focus on improving the feeding and rearing protocol until 30 DAH.

## 1. Introduction

The freshwater eel *Anguilla japonica* is a temperate catadromous fish distributed in the Philippines, Taiwan, China, Japan, and Korea [1,2,3]. This is a commercially important species in Northeast Asia, including Korea. However, eel aquaculture is dependent on the supply of seedlings (juveniles) through natural catches. The supply has been decreasing due to overfishing, habitat destruction, and changes in the marine environment [4]. Therefore, mass production of cultured seed is necessary to stabilize the eel aquaculture industry and preserve the species.

Research concerning cultured freshwater eel seed production began in the 1960s [5]. Yamamoto and Yamauchi [6] first succeeded in producing fertilized eggs using hormone treatments and reared larvae for up to five days after hatching (DAH). Tanaka et al. [7] later succeeded in growing larvae to the glass eel stage (seedling, juvenile), after which researchers succeeded in closing the eel life cycle by producing second-generation eels in 2010 [8,9,10]. However, artificial eel seed production was performed in laboratory-scale tanks and has not yet been developed on a commercial scale.

At present, due to the low production and high cost of cultured eel larvae in the laboratory, cultured eel seed is not used in the eel aquaculture industry [11]. The low production is due to slow growth by nutritional problems [11] and to a very low survival rate of less than 1% [12]. Moreover, the high cost is caused by the time and labor costs of feeding at high frequencies and replacing rearing tanks every day due to the characteristics of the slurry type diet and the small scale rearing tank. Therefore, for efficient eel mass seed production, a rearing and feeding protocol optimized for eel larvae is required.

One of the major reasons for the difficulty in developing an optimized rearing method despite a long period of active research is related to the unique and long early life history of the freshwater eel. *A*. *japonica* inhabits freshwaters in Northeast Asia and migrates to the seamount of the West Mariana Ridge to spawn and hatch at depths of 150–200 m [13]. The eel larvae feed on marine snow (i.e., particulate organic matter, such as zooplankton fecal pellets and discarded larvacean houses) as a food source [14,15,16] and migrate to the Eddy region near Taiwan by the North Equatorial Current and the Kuroshio Current, where they metamorphose into the juvenile (glass eel) stage and migrate from the sea to rivers [17]. The period of metamorphosis from larva to juvenile occurs at 110–170 DAH in wild [18,19] and at more than 200–250 DAH in culture [20]. Therefore, the freshwater eel differs from the early life history of common marine fish, and the details of its early life history remain to be elucidated.

For successful artificial seed production, it is important to develop an appropriate rearing protocol according to the developmental stage through understanding the early life history. In particular, the morphology during the larval stage can reflect the ecophysiology as a result of adaptation to the habitat. Fish larvae prioritize the growth of organs involved in primary functions (i.e., feeding, respiration, and locomotion) rather than organs with lower priorities for growth and survival, and the changes in body form occur due to the differential growth of organs [21,22]. Knowledge regarding these relative growth patterns during early development can contribute to larviculture by characterizing normal growth patterns under certain conditions and optimizing rearing protocols if abnormalities in larval development are detected [23,24,25,26]. Therefore, to improve the eel mass seed production technology, it is important to understand the growth characteristics of the larval stage in a culture environment.

There have been a number of previous studies on the morphology and growth characteristics of eel larvae. Mochioka [27] reported the morphology and growth characteristics of larvae from 10 to 60 mm in total length (TL) in wild. Although there have been many studies related to the morphology of eel larvae under culture conditions, such as the morphology and TL during 0–8 DAH [28], the morphology, TL, and preanal length during 0–15 DAH [29], the morphology, TL, head length, and trunk height during 0–100 DAH [30], the morphology during 100–270 DAH [7], the morphology during 0–268 DAH [31], TL and trunk height at 25 and 55 DAH [32], TL during 0–80 DAH [11], distribution of TL at 96 and 215 DAH [33], and the morphology, TL, and preanal length during metamorphosis [34,35,36], there have been no previous studies regarding morphological development and allometric growth patterns during long-term.

The aim of the present study was to describe the morphological development and allometric growth patterns for *A*. *japonica* larvae reared under culture conditions. In addition, we tried to confirm the growth characteristics of each larval stage and to understand the relationship between changes in allometric growth patterns and early mass mortality. Finally, we provided the basic data necessary to understand the early life history of *A*. *japonica* and to improve artificial seed rearing technology.

## 2. Materials and Methods

### 2.1. Fish

This study was conducted at the National Institute of Fisheries Science in Sirang-ri, Gijang-eup, Gijang-gun, Busan, South Korea. The rearing water was adjusted to the target temperature with a heat pump system (DHA-500-SLCF; Daeil Systems, Yongin-si, Gyeonggi-do, South Korea) after passing natural seawater through a high-pressure sand filter, ultraviolet filter, and 25- and 10-μm cartridge filters. 28 female freshwater eels collected in September 2018 in Buan, Jeollanam-do, South Korea, and males reared in a fish farm for two years were used for seed production. Parental fishes were allowed to slowly acclimatize to the rearing water at 20 °C, with an increase of 5 psu/day to 35 psu, for 1 week. For identification, microchips (Taechang, Seoul, South Korea) were inserted into the back muscles, and each fish was identified, tracked, and managed using a hand reader (RT160; Fofia, Wuxi, China). To induce maturity in parental fishes according to Kim et al. [29], 20 mg/fish of salmon pituitary extract (SPE; Yasaka, Abashiri, Hokkaido, Japan) were injected into the abdominal cavity of females weekly. A total of 24 females matured after an average of 7.92 weeks. For males, human chorionic gonadotropin (HCG; Daesung Microbiological Labs, Uiwang-si, Gyeonggi-do, South Korea) was injected into the abdominal cavity at 1 IU/g (body weight) for 12 weeks. Eggs were obtained by cannulation when the female body weight increased by >20% compared to the initial body weight. When the diameter of the obtained eggs reached > 900 μm, maturity and ovulation were induced in females by injection of 10 mg/fish of 17α-hydroxyprogesterone (OHP; Sigma-Aldrich, St. Louis, MO, USA) and 10 mg/fish of 17α,20β-dihydroxy-4-pregnen-3-one (DHP; Sigma-Aldrich, St. Louis, MO, USA) into the abdominal cavity. Males were induced to ejaculate by abdominal compression, and sperm activity was checked using a Computer-Assisted Sperm Analysis (CASA) system (IVOS-II; Hamilton Throne Research, Beverly, MA, USA). The individual with the highest mean sperm activity was selected for breeding. Of the total 28 females, 17 females laid eggs, of which 10 batches hatched and only 7 batches survived until 7 DAH. Among them, 5 batches survived until an average of 37.8 DAH, and only 2 batches metamorphosed into glass eel. However, one batch could not be analyzed since only 20 larvae remained at 100 DAH. So, morphological and allometric changes were analyzed for one batch. As a result, to produce fertilized eggs, a mature female (initial 1042 g) and the selected male (initial 187 g) were combined in an ovulation induction tank with 20 °C seawater at 17:00 on 26 December 2018, and the seawater was slowly warmed to 23 °C. At 09:00 on 27 December 2018, the naturally laid fertilized eggs were collected in an egg collection tank. The floating fertilized eggs were transported to an incubation tank at 23 °C, gently aerated, and managed until hatching after approximately 48 h.

### 2.2. Larvae Rearing

Hatched larvae were housed in a 500-L vertical cylindrical tank at approximately 500 individuals/L, and reared in running water at 23 °C supplied at 2 L/min without feeding until 6 DAH. Eel larvae at 6 DAH were transferred to a 20-L round acrylic resin tank at approximately 100 individuals/L, and the first feeding was performed at 7 DAH at 15:00. The feed based on the eggs of spiny dogfish consisted a slurry of soybean meal, fish meal, krill meal, and vitamins in accordance with the method established by Kim et al. [37] (Table 1). The water flow rate and feed viscosity were approximately 1.0 L/min and 100 mPa·s from 7 to 50 DAH, respectively. The feed (6 mL) was supplied five times daily (09:00, 11:00, 13:00, 15:00, and 17:00) on the bottom of each tank. The water flow was stopped since eel larvae are not good swimmers when feeding. The water flow and feed viscosity were supplied at 1.0 L/min and 100 mPa·s on 7 DAH and increased gradually to 1.5 L/min and 200 mPa·s on 200 DAH with growth.

### 2.3. Survival Rate

To confirm the survival rate of eel larvae during ontogenesis, 500 eel larvae on 7 DAH were kept in eight 20-L round acrylic resin tanks. The rearing protocol was as described in Section 2.2 above. All eel larvae were counted at 15, 30, 50, 70, and 100 DAH. After keeping 28 larvae in 14 tanks on 100 DAH, all eel larvae were counted at 100, 125, 150, 175, and 200 DAH to confirm the changes in survival rate over time.

### 2.4. Sampling

**Figure 1:**
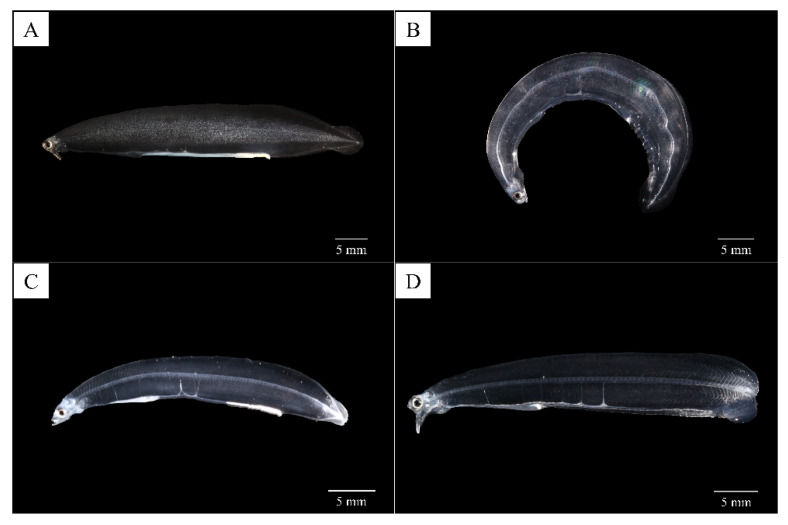
Abnormality of the *Anguilla japonica* in the leptocephalus stage in culture. (**A**) Posterior bent lower jaw. (**B**) Dorsal curvature of the notochord column. (**C**) Decayed tail fin. (**D**) Vertebrae compression and fusion of the tail region.

Eel larvae with no abnormalities (20 larvae × 18 sampling points) were sampled at 0–7, 10, 15, 30, 50, 70, 100, 125, 150, 175, and 200 DAH (Figure 1). After anesthetizing with 200 ppm ethyl 3-aminobenzoate methane sulfonate (MS-222; Sigma-Aldrich, St. Louis, MO, USA), larvae during 0–50 DAH were photographed using a digital camera (AxioCam MRc5; Carl Zeiss, Oberkochen, Germany) connected to a stereomicroscope (SteREO Discovery V20; Carl Zeiss, Oberkochen, Germany), and larvae during 70–200 DAH were photographed using a digital camera (EOS 5D Mark II; Canon, Tokyo, Japan).

### 2.5. Morphometric Analysis

**Figure 2:**
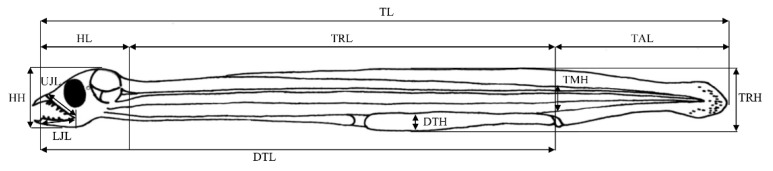
Morphometric measurements in *Anguilla japonica* larvae. Abbreviations: DTH, digestive tract height; DTL, digestive tract length; HH, head height; HL, head length; LJL, lower jaw length.; TAL, tail length; TL, total length; TMH, tail muscle height; TRH, trunk height; TRL, trunk length; UJL, upper jaw length.

The TL, head length, head height, trunk length, trunk height, tail length, tail muscle height, digestive tract length (equal preanal length), digestive tract height, upper jaw length, lower jaw length, yolk sac area, oil globule area, and intestine area were measured on photographs of larvae using image analysis software (ZEN v. 2012; Carl Zeiss, Oberkochen, Germany) (Figure 2).

### 2.6. Allometric Analysis

Allometric growth during the larval stages was calculated as a power function of TL, using the data of the measured morphometric characteristics with the following model:

*y* = *ax^b^*,

where *y* is the measured character, *a* is the intercept, *x* is the TL, and *b* is the growth coefficient [21]. With regard to the specific morphological characteristics, the height was compared with TL and isometric growth was determined when *b* = 1 for length. Positive allometric growth was determined when *b* > 1, corresponding to a higher growth rate than TL, while negative allometric growth was determined when *b* < 1. Linear regression analysis was performed on the log transformed data according to log*y* = log*a* + *b*log*x* [38]. Regression lines were calculated for *x*_min_ to *x*_intermediate_, and for *x*_intermediate_ to *x*_max_ and the growth coefficients from both linear regression equations were compared using the *t* test (*p* < 0.01) [39]. The inflection point was calculated as *x*_intermediate_ when it showed the largest *t* value and the regression lines had *p* < 0.05.

### 2.7. Statistical Analysis

Data are shown as the mean ± standard deviation (SD). Potential differences in yolk sac area and oil globule area were tested by one-way analysis of variance (ANOVA). The null hypothesis is that there are no differences among 0–7 DAH. When a significant difference was found, Tukey’s honestly significant difference (HSD) test was performed. An independent two-sample *t* test was performed to compare the mean values between upper jaw length and lower jaw length. The null hypothesis is that there are no differences between upper jaw length and lower jaw length. In all analyses, *p* < 0.05 was taken to indicate statistical significance. Statistical analyses were performed and graphics were prepared using SPSS software, version 25 (IBM, Armonk, NY, USA) and Excel 2016 software (Microsoft, Redmond, WA, USA).

## 3. Results

### 3.1. Identification of Larval Stages 

**Figure 3:**
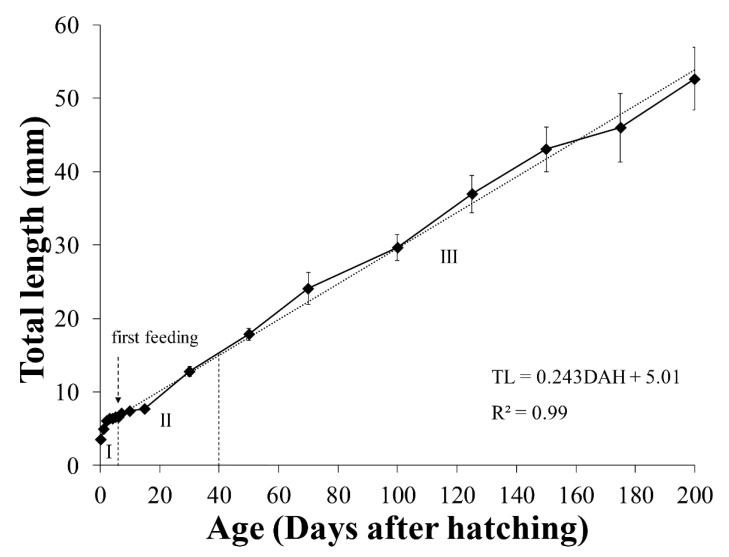
Growth of total length in *Anguilla japonica* from 0 to 200 DAH (stage I: yolk sac larvae; stage II: pre-leptocephalus; stage III: leptocephalus). Values are means ± SD. Abbreviations: DAH, days after hatching; TL: total length.

In this study, the initial average size of *A. japonica* larvae was 3.58 ± 0.15 mm TL and increased following TL = 0.2419DAH + 5.016 (R^2^ = 0.99), reaching 52.66 ± 4.26 mm at 200 DAH. According to the morphological criteria of wild eel larvae reported previously [27], the eel larval stages under culture conditions were characterized as follows: yolk sac larval stage (from after hatching until the presence of endogenous reserves), pre-leptocephalus stage (needle-like teeth, the anus has moved to the posterior of the body, and the number of preanal myomeres has increased), and leptocephalus stage (teeth replaced by regular broad-based teeth, the position of the anus remains stable). According to the morphological criteria, the yolk sac larval stage was from 0 to 6 DAH, pre-leptocephalus stage was from 7 to 30 DAH, and leptocephalus stage was from 50 to 200 DAH. To separate the eel larval stages by TL, yolk sac larvae stage and pre-leptocephalus stage were divided by the average TL between 6 DAH (6.59 mm) and 7 DAH (7.10 mm), and pre-leptocephalus stage and leptocephalus stage were divided by the average TL between 30 DAH (12.77 mm) and 50 DAH (17.86 mm). The TL of the characterized larval stages were as follows: yolk sac larvae stage (larval stage I: 0–6 DAH, 3.23–6.85 mm TL), pre-leptocephalus stage (stage II: 7–30 DAH, 6.85–15.31 mm TL), and leptocephalus stage (stage III: 50–200 DAH, 15.31–60.06 mm TL) (Figure 3).

### 3.2. Survival Rate

**Figure 4:**
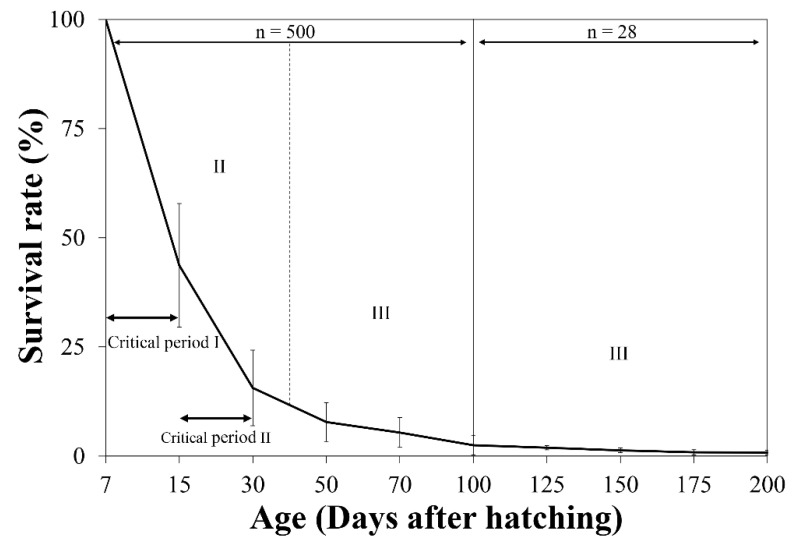
Survival rates of *Anguilla japonica* larvae during three larval stages from 7 to 200 DAH. Values are means ± SD. Abbreviations: DAH, days after hatching; II, stage II (pre-leptocephalus stage); III, stage III (leptocephalus stage).

The survival rate of eel larvae decreased sharply after the first feeding. The survival rate at 15 DAH was 43.70 ± 14.15%. The highest mortality rate was recorded at this time point, which was determined to be critical period I. The survival rate at 30 DAH was 15.60 ± 8.66%, and the second highest mortality rate was recorded at this time point, which was determined to be critical period II. The survival rate continued to decrease during 50–200 DAH (7.78 ± 4.44% at 50 DAH, 2.48 ± 2.18% at 100 DAH, 1.30 ± 0.54% at 150 DAH, and 0.79 ± 0.50% at 200 DAH). Statistical analysis was not performed on the survival data since the number of specimens decreased during the experiment rearing (Figure 4).

### 3.3. Morphological Development

#### 3.3.1. Yolk Sac Larvae (Stage I; 0–6 DAH)

**Figure 5:**
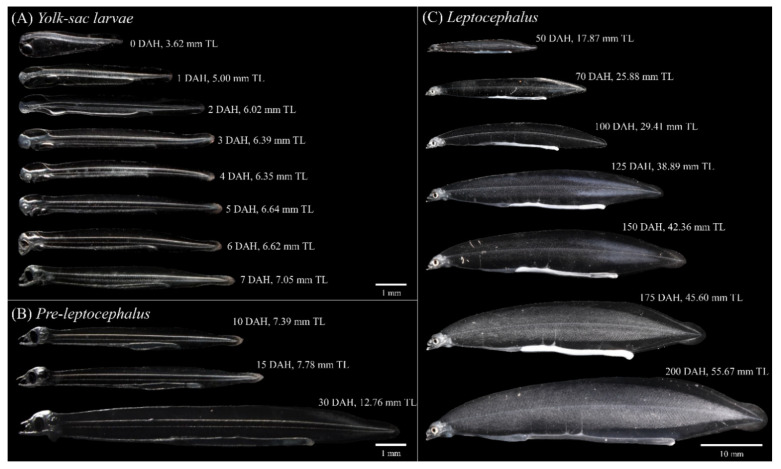
Morphological development in early ontogeny of *Anguilla japonica* larvae. (**A**) Yolk sac larval stage. (**B**) Pre-leptocephalus stage. (**C**) Leptocephalus stage. Abbreviations: DAH, days after hatching; TL, total length.

**Figure 6:**
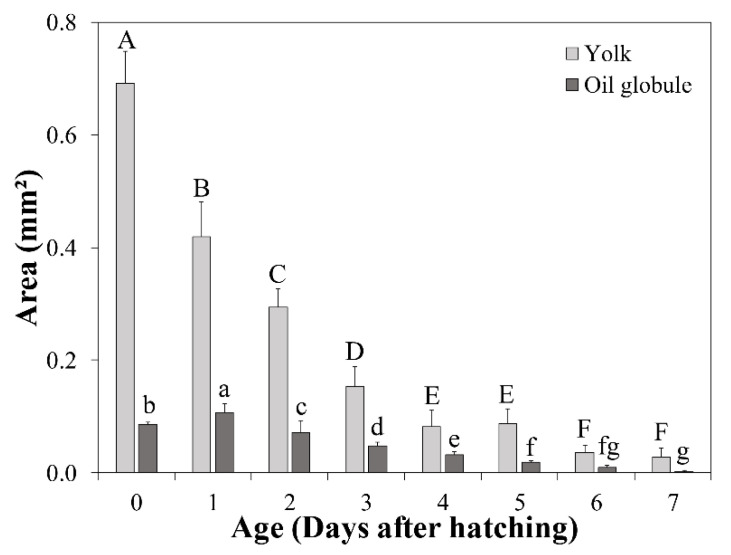
Yolk sac and oil globule utilization in *Anguilla japonica* larvae. Values are means ± SD. Different uppercase letters denote significant differences between yolks (light gray). The significant differences between oil globules (dark gray) are shown using different lowercase letters (*p* < 0.05).

**Figure 7:**
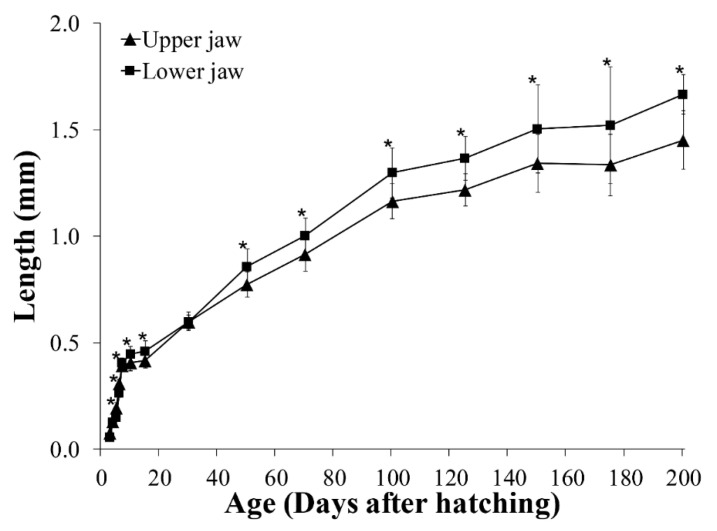
Upper and lower jaw lengths in *Anguilla japonica* larvae. Values are means ± SD. Asterisks indicate significant differences between upper and lower jaw lengths (*p* < 0.05).

At hatching, the average TL of *A. japonica* larvae was 3.58 ± 0.15 mm, and the larvae had a large yolk sac (area = 0.691 ± 0.057 mm^2^) containing one oil globule (area = 0.086 ± 0.004 mm^2^) in the lower anterior part. The body was transparent, and several melanophores were observed at the end of the notochord. At 1 DAH, the average TL was 5.01 ± 0.20 mm, the oil globule was pushed back as the front yolk sac was absorbed, and a yolk syncytial layer appeared at the point of yolk absorption. Melanophores moved to the tip of the caudal fin. At 3 DAH, the average TL was 6.41 ± 0.21 mm, the mouth and anus were open, and a straight digestive tract was observed along the notochord. The area of the yolk sac decreased significantly until 4 DAH, when the body changed into a slender type. At 5 DAH, the average TL of *A. japonica* larvae was 6.65 ± 0.21 mm, and a canine-shaped front tooth appeared. There were no significant differences in length between the upper and lower jaws before 5 DAH (*p* > 0.05), but at 5 DAH, significant differences began to be observed between the upper jaw length (0.151 ± 0.017 mm) and lower jaw length (0.195 ± 0.026 mm) (*p* < 0.05). At 6 DAH, the average TL was 6.59 ± 0.18 mm, the eyes were darkly pigmented, and more melanophores were observed at the tip of the caudal fin with growth. The angle of the anterior tip of the upper jaw to the esophagus was approximately 160° and was turned downward, and a number of needle-like teeth were observed. There was a significant difference between upper jaw length (0.310 ± 0.019 mm) and lower jaw length (0.265 ± 0.022 mm) (*p* < 0.05). The average the yolk area was 0.036 ± 0.013 mm^2^, and a significant difference was confirmed at 5 DAH (0.087 ± 0.027 mm^2^) (*p* < 0.05). However, there was no significant difference in the average yolk area between 6 and 7 DAH (0.029 ± 0.016 mm^2^) (*p* > 0.05), so absorption of the yolk sac was determined to be complete at 6 DAH. The oil globule was mostly absorbed at 7 DAH (0.002 ± 0.002 mm^2^), so the completion of oil globule absorption was also determined to be at 6 DAH. Therefore, the yolk sac larval stage was characterized by completion of the absorption of endogenous reserves (Figure 3, Figure 5A, Figure 6 and Figure 7). 

#### 3.3.2. Pre-Leptocephalus (Stage II; 7–40 DAH)

At 7 DAH, the average TL was 7.10 ± 0.25 mm, and lower jaw length (0.407 ± 0.021 mm) was significantly longer than the upper jaw length (0.394 ± 0.024 mm) (*p* < 0.05). With growth of the lower jaw, the angle of the anterior tip of the upper jaw to the esophagus became parallel to approximately 180°, and eel larvae began exogenous feeding. Melanophores increased in number at the tip of the caudal fin, and the eyes became darker with more pigmentation. Digestive tract length (preanal length) was 4.93 ± 0.15 mm, and the anus was located at 69.4% of the body length. At 15 DAH, the average TL was 7.73 ± 0.43 mm, and the lower jaw length (0.460 ± 0.049 mm) remained significantly longer than the upper jaw length (0.417 ± 0.035 mm) (*p* < 0.05). In addition, the rectum valve developed and began to differentiate between the intestine and the rectum. Digestive tract length (preanal length) was 5.84 ± 0.30 mm, and the anus was located at 75.5% of the body length, and the number of preanal myomeres was 59–61 (*n* = 5). At 30 DAH, the average TL was 12.77 ± 0.66 mm, and the needle-like teeth had decreased in size. There was no significant difference between upper jaw length (0.597 ± 0.034 mm) and lower jaw length (0.600 ± 0.044 mm) (*p* > 0.05). A small number of melanophores were present at the tip of the caudal fin. Digestive tract length (preanal length) was 10.15 ± 0.54 mm, and the anus was located at 79.5% of the body length. The number of preanal myomeres was 63–71 (*n* = 5), which had increased from 15 DAH. Therefore, the pre-leptocephalus stage was characterized by needle-like teeth and an increase in number of preanal myomeres with the anus moved to the posterior of the body (Figure 3, Figure 5B and Figure 7).

#### 3.3.3. Leptocephalus (Stage III; 40–200 DAH)

At 50 DAH, the average TL was 17.86 ± 0.82 mm, and most of the melanophores at the tip of the caudal fin had disappeared. The needle-like teeth were replaced by regular broad-based teeth, and a significant difference was observed between upper jaw length (0.773 ± 0.058 mm) and lower jaw length (0.859 ± 0.081 mm) (*p* < 0.05). The digestive tract length (preanal length) was 14.02 ± 0.54 mm, and the position of the anus was 78.5% of the body length. The number of preanal myomeres was 69–73 (*n* = 5), which was greater than at 30 DAH. At 100 DAH, the average TL was 29.65 ± 1.75 mm, and digestive tract length (preanal length) was 22.03 ± 1.36 mm. The anus was located at 74.3% of the body length, and the number of preanal myomeres was 72–73 (*n* = 5). At 150 DAH, the average TL was 43.07 ± 3.05 mm, and with the continuous increase in trunk length, the body shape changed to a willow leaf-like form. Digestive tract length (preanal length) was 31.57 ± 2.19 mm, the anus was located at 73.3% of the body length, and the number of preanal myomeres was 72–73 (*n* = 5). At 200 DAH, the average TL was 52.66 ± 4.26 mm, and a significant difference between upper jaw length (1.452 ± 0.139 mm) and lower jaw length (1.667 ± 0.092 mm) was observed continuously from 50 to 200 DAH (*p* < 0.05). Digestive tract length (preanal length) was 38.47 ± 2.83 mm, the position of the anus decreased to 73.0% of body length with growth, and the numbers of preanal myomeres was 71–73 (*n* = 5). Therefore, in leptocephalus stage, eel had the characteristic that needle-like teeth were replaced by regular broad-based teeth, the position of the anus moved forward, and the number of preanal myomeres was maintained at 71–73 (Figure 3, Figure 5C and Figure 7).

### 3.4. Allometric Growth

#### 3.4.1. Larval Stages

**Figure 8:**
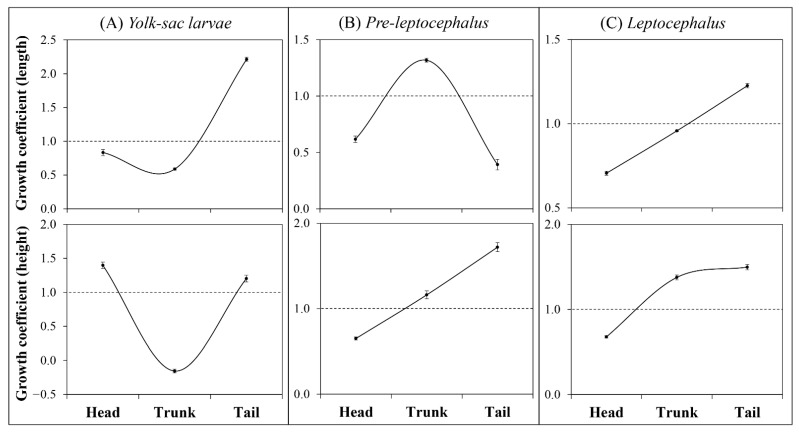
Growth coefficients of head, trunk, and tail parts in larval stages of *Anguilla japonica*. (**A**) Yolk sac larval stage: 3.23–6.85 mm TL. (**B**) Pre-leptocephalus stage: 6.85–15.31 mm TL. (**C**) Leptocephalus stage: 15.31–60.06 mm TL. Values are means ± SD. Abbreviation: TL, total length.

In the yolk sac larval stage, head height and tail parts (length and height) showed positive allometric growth (*b* = 1.40, *b* = 2.21, and *b* = 1.20, respectively). However, head length and trunk parts (length and height) showed negative allometric growth (*b* = 0.83, *b* = 0.59, and *b* = −0.16, respectively) (Figure 8A). In the pre-leptocephalus stage, head parts (length and height) showed negative allometric (*b* = 0.62 and *b* = 0.65, respectively), while trunk parts (length and height) showed positive allometric growth (*b* = 1.32 and *b* = 1.16, respectively). The tail parts showed negative allometric growth in length (*b* = 0.39) and positive allometric growth in height (*b* = 1.72) (Figure 8B). In the leptocephalus stage, head parts (length and height) showed negative allometric growth (*b* = 0.71 and *b* = 0.68, respectively). In the trunk parts, length showed nearly isometric growth (*b* = 0.96) and height showed positive allometric growth (*b* = 1.38). Tail parts (length and height) showed positive allometric growth (*b* = 1.23 and *b* = 1.50, respectively) (Figure 8C).

#### 3.4.2. Body Parts

**Figure 9:**
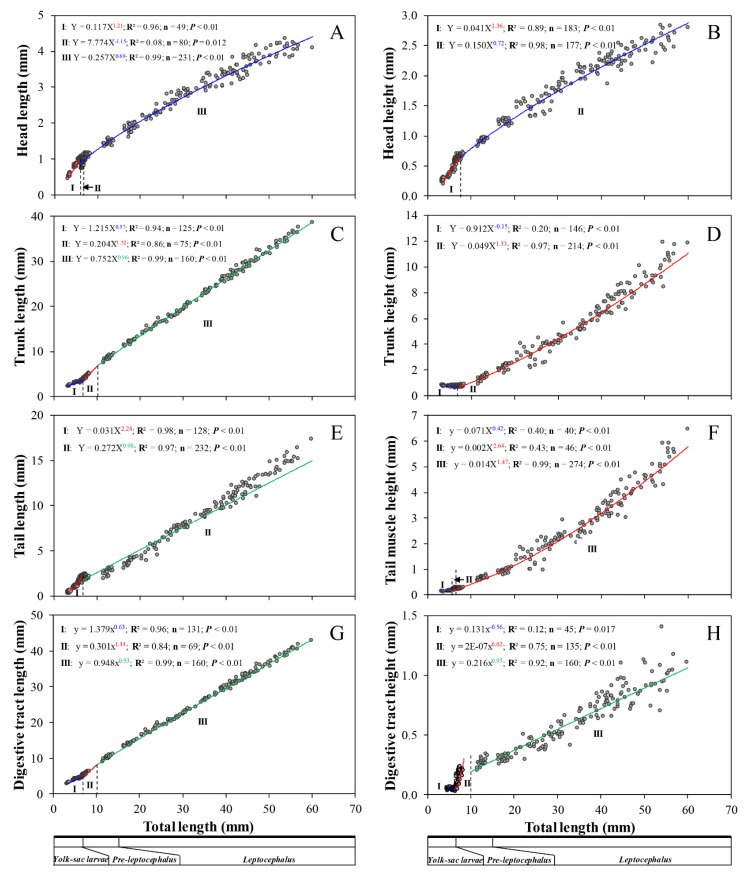
Allometric growth equations between measured body proportions from 0 to 200 DAH in *Anguilla japonica* larvae. (**A**) Head length. (**B**) Head height. (**C**) Trunk length. (**D**) Trunk height. (**E**) Tail length. (**F**) Tail muscle height. (**G**) Digestive tract length. (**H**) Digestive tract height. The dashed lines represent the inflexion points of growth. Abbreviation: DAH, days after hatching.

Growth of head length was divided into three distinct phases in ontogeny of eel larvae. Phase I showed positive allometric growth during 3.23–5.99 mm TL (*a* = 0.117, *b* = 1.21, R^2^ = 0.96). In contrast, phase II and showed negative allometric growth with the head decreasing during 5.99–6.75 mm TL (*a* = 7.774, *b* = −1.15, R^2^ = 0.08) and during 6.75–60.06 mm TL (*a* = 0.257, *b* = 0.69, R^2^ = 0.99), respectively (Figure 9A). Growth of head height was divided into two distinct phases. Phase I involved positive allometric growth during 3.23–7.57 mm TL (*a* = 0.041, *b* = 1.36, R^2^ = 0.89), while phase II showed negative allometric growth during 7.57–60.06 mm TL (*a* = 0.150, *b* = 0.72, R^2^ = 0.98) (Figure 9B). As a result, head parts showed positive allometric growth in the early stages and the growth pattern then changed to negative allometric growth. Growth of trunk length was divided into three distinct phases. Phase I involved negative allometric growth during 3.23–6.69 mm TL (*a* = 1.215, *b* = 0.57, R^2^ = 0.94), while phase II showed positive allometric growth during 6.69–10.01 mm TL (*a* = 0.204, *b* = 1.52, R^2^ = 0.86), and thereafter the growth trend became near isometric during 10.01–60.06 mm TL (*a* = 0.752, *b* = 0.96, R^2^ = 0.99) (Figure 9C). Growth of tail length was divided into two distinct phases. Phase I involved positive allometric growth during 3.23–6.72 mm TL (*a* = 0.031, *b* = 2.24, R^2^ = 0.98), while phase II showed near isometric growth during 6.72–60.06 mm TL (*a* = 0.272, *b* = 0.98, R^2^ = 0.97) (Figure 9E). Therefore, the lengths of both the tail and trunk showed characteristic near isometric changes. Growth of trunk height was divided into two distinct phases. Phase I showed negative allometric growth during 3.23–6.90 mm TL (*a* = 0.912, *b* = −0.15, R^2^ = 0.20), while phase II showed positive allometric growth during 6.90–60.06 mm TL (*a* = 0.049, *b* = 1.33, R^2^ = 0.97) (Figure 9D). Growth of tail muscle height was divided into three distinct phases. Phase I showed negative allometric growth during 3.23–5.50 mm TL (*a* = 0.071, *b* = 0.42, R^2^ = 0.40), while phases II showed positive allometric growth during 5.50–6.43 mm TL (*a* = 0.002, *b* = 2.64, R^2^ = 0.43), and phase III also showed positive allometric growth during 6.43–60.06 mm TL (*a* = 0.014, *b* = 1.47, R^2^ = 0.99) (Figure 9F). Thus, the heights of both the tail and trunk finally showed positive allometric growth. The changes in length and height of the digestive tract were divided into three distinct phases. Digestive tract length showed negative allometric growth during 3.23–6.78 mm TL (*a* = 1.379, *b* = 0.63, R^2^ = 0.96), positive allometric growth during 6.78–10.01 mm TL (*a* = 0.301, *b* = 1.44, R^2^ = 0.84), and became near isometric during 10.01–60.06 mm TL (*a* = 0.948, *b* = 0.93, R^2^ = 0.99) (Figure 9G). Similarly, digestive tract height showed negative allometric growth during 4.64–6.26 mm TL (*a* = 0.131, *b* = −0.56, R^2^ = 0.12), positive allometric growth during 6.26–10.01 mm TL (*a* = 2 × 10^−7^, *b* = 6.62, R^2^ = 0.75), and finally became near isometric during 10.01–60.06 mm TL (*a* = 0.216, *b* = 0.95, R^2^ = 0.92) (Figure 9H). These observations indicted that the growth patterns of digestive tract parts changed in the order of negative allometric, positive allometric, and isometric growth.

## 4. Discussion

The fish larvae stage represents the stage with the highest vulnerability in a fish life cycle [40,41,42]. Initial mass mortality occurs internally due to the drastic development of organs, including the digestive and swimming organs necessary for the primary nutrient source shifts from endogenous reserve to exogenous feeding [43] and externally due to starvation and predation [44]. Therefore, in order to increase seed production in a culture environment, it is important to provide an optimal rearing protocol with an understanding of the change of internal factors.

In this study, the size of TL that separated the larval stages was similar to the sizes dividing wild eel larval stages reported by Mochioka [27], namely yolk sac larval stage (until approximately 7 mm) and pre-leptocephalus stage (until approximately 15 mm). However, Mochioka [27] reported that the number of preanal myomeres was maintained at approximately 79 and the position of the anus remained stable in the leptocephalus stage, while in the present study the number of preanal myomeres was maintained at approximately 72 and the anus moved to the anterior of the body. A previous study also reported that the number of preanal myomeres in cultured eel larvae was lower until 100 DAH [30]. Wild eel larvae are known to grow at a rate of 0.5 mm/day [11,45], while the growth rate of cultured eel larvae in this study (0.2–0.3 mm/day) was only half that of wild larvae. Cultured eel larvae were reported previously to show similar growth rates until 100 DAH [30]. Therefore, cultured and wild eel larvae showed differences in the number of preanal myomeres and growth rate, which were thought to be due to artificial maturity, feed, and rearing environment. Further studies of the reasons for these differences are warranted.

In this study, the average yolk sac area after hatching was 0.691 ± 0.057 mm^2^. Larvae of the European eel, *Anguilla anguilla*, which belongs to the same genus as *A. japonica*, were reported to show a similar yolk sac area of 0.6–0.7 mm^2^ at 22 °C [46]. The average oil globule area of *A. japonica* larvae after hatching was 0.086 ± 0.004 mm^2^, but increased to 0.107 ± 0.016 mm^2^ at 1 DAH in the present study. This was assumed not to be an actual increase in size of the oil globule, but to have been due to the fact that the oil globule was moved to the back as the yolk was rapidly absorbed and was pressed, resulting in an increased measurement. Indeed, at 1 DAH, the average yolk sac area had decreased by 39.3% and the average trunk height had also decreased by 0.043 mm. In this study, the endogenous reserves of eel larvae were mostly absorbed before 7 DAH, and the first feeding started at 7 DAH, so they had a very short period of mixed feeding or no mixed feeding period. The mixed feeding period is a period with the simultaneous consumption of endogenous and exogenous feeding [47], which helps the growth and survival of the larvae by temporary nutritional supplementation during the transition to exclusively exogenous feeding and prevents nutritional deficiencies during starvation [48]. In some teleost fishes, including the tiger grouper (*Epinephelus fuscoguttatus*), Asian sea bass (*Lates calcarifer*), and olive flounder (*Paralichthys olivaceus*), short starvation or immediate feeding after the completion of endogenous reserve absorption has been shown to result in better growth and survival [49,50,51]. Therefore, eel larvae may also show increased growth and survival if fed at the optimal initial feeding time. In particular, as starvation can easily occur in eel larvae due to the characteristics of the slurry-type diet under culture rearing conditions [52], good management and appropriate rearing protocols specific to eel are necessary.

As shown in Figure 1A, numerous eel larvae feeding on the slurry-type diet with the posteriorly bent lower jaw were observed in the leptocephalus stage. This abnormality was thought to be due to elongation of the lower jaw and increased swimming ability with growth during the process of feeding the slurry-type diet supplied at the bottom of the tank. Indeed, from 50 DAH, there was a continuous significant difference between the average length of the upper jaw and the lower jaw (*p* < 0.05), and at 50 DAH, the length of the lower jaw was longer by only 0.088 mm, but the difference increased with growth, and it became longer by 0.215 mm at 200 DAH. These eel larvae with lower jaw deformity showed similar growth and survival compared to normal larvae during the larval stage feeding the slurry-type diet, but they did not survive longer since they were fed with the pellet or paste-type diet after metamorphosis into glass eels (juveniles). Therefore, to prevent deformity of the lower jaw of eel larvae, it is necessary to develop a new non-slurry-type diet suitable for the leptocephalus stage.

Allometry is related to the chronology of important early life history events [24]. Larvae adjust the development rate of certain structures according to the physiological priorities of the species for survival [53]. In general, allometric growth of larval body regions shows a U-shaped growth profile (i.e., allometric growth of head and tail regions exhibiting higher growth rates compared to the trunk region) during the endogenous feeding period [21,24,25,54,55,56]. In this study, in the yolk sac larval stage, allometric growth of the larval head parts (length and height) showed a U-shaped growth profile. This growth pattern could be interpreted as an adaptive strategy to reduce and optimize the energy cost of larval transport [22,56,57]. During the early larval stage, the head parts commonly exhibited positive allometric growth, which is an early developmental strategy of teleost fish to give priority to development of the brain, respiratory, sensory, and feeding organs [24,25,26,54,55]. In this study, head length showed negative allometric growth (*b* = 0.83) in the yolk sac larval stage. Indeed, head length of eel larvae showed positive allometric growth (*b* = 1.21) from hatching to 5.99 mm TL (approx. 2 DAH), which then changed to negative allometric growth (*b* = −1.15). This growth pattern of head length was thought to be due to the change in position of the top of head. The top of the head of eel larvae faces forward at hatching. However, with the development of the lower jaw, the top of the head gradually changes to face upward, so eel larvae measurements appear to show a decrease in head length from 3 DAH (1.003 ± 0.035 mm) to 6 DAH (0.767 ± 0.031 mm). Therefore, eel larvae are considered to have advantages in the brain, respiratory, sensory, and feeding organs with prioritization of the development of head parts, similar to the early developmental strategy of teleost fish. On the other hand, eel larval head parts (length and height) continued to show negative allometric growth. In many teleost species, the pattern of allometric growth tends to change to isometric growth during transformation to the juvenile stage [21,57]. However, the head parts of eel larvae maintained negative allometric growth until before metamorphosis stage, which was assumed to be related to maintenance of the willow leaf-like body shape of eel larvae.

In general, the growth pattern of eel larval trunk parts is thought to be related to the digestive system, but as eel larvae have a straight rather than a coiled gut, it would be more reasonable to understand in terms of body shape change than in relation to the digestive system. In this study, in the yolk sac larval stage, trunk parts (length and height) showed negative allometric growth and tail parts (length and height) showed positive allometric growth, similar to a U-shaped growth profile of general teleost species. Subsequently, in the pre-leptocephalus stage, trunk length changed to show positive allometric growth (*b* = 1.318) and tail length to negative allometric growth (*b* = 0.392). However, as the fiducial line of the trunk and tail is divided at the position of the anus, this change in growth pattern was considered to be a measurement error due to the change in position of the anus with growth. Indeed, as the position of the anus tends to move posteriorly in eel larvae during the pre-leptocephalus stage and to move anteriorly during the leptocephalus stage, it would be appropriate to check the allometric growth of trunk and tail in terms of height rather than length to understand the growth characteristics of eel larvae. 

In terms of height, both the trunk and tail showed positive allometric growth in the pre-leptocephalus stage and leptocephalus stage. In addition, when the inflection point of the allometric growth pattern was confirmed, trunk height showed negative allometric growth (*b* = −0.15) due to the decrease of yolk until 6.900 TL mm (6–7 DAH), and then showed positive allometric growth until the end of the experiment. Tail muscle height showed negative allometric growth (*b* = 0.42) up to 5.501 mm TL (1–2 DAH), with rare swimming but high positive allometric growth (*b* = 2.64) until 6.425 mm TL (approx. 4 DAH) before the first feeding. Thereafter, continuous positive allometric growth (*b* = 1.47) was observed until the end of the experiment. As mentioned above, allometric growth of the larvae of teleost species becomes close to isometric growth with growth to the juvenile stage, but eel larvae showed continuous positive allometric growth in trunk and tail height and continuous negative growth in their head parts. These growth patterns were assumed to reflect the unique early life history of eels. The eel has a unique willow leaf-like form during its early life history, and these growth patterns of eel larvae were also thought to be related to the body shape. The willow leaf-like form body of leptocephalus is suitable for long distance dispersal by currents [1], and may have been adapted to the characteristics of the early ecology involving migration over long distances. Positive allometric growth of tail muscle height is known to contribute to improvement of swimming ability [26]. As *A*. *japonica* leptocephali is known to have a diel vertical migration, staying at 100 m at night and migrating deeper during the daytime [58]. Most leptocephali species with visible gut contents appear to be more frequently caught during the day and crepuscular periods [59,60], which may be a strategy to facilitate digestion and assimilation of food and growth in the warmer water after feeding occurs in deeper layers [61,62,63]. So, the positive allometric growth of tail muscle height is thought to reflect the early ecology of eels. Overall, these growth patterns of trunk height, tail muscle height, and head parts may be adaptive characteristics for the unique early life history of *A. japonica*.

In this study, the digestive tract parts (length and height) of eel larvae showed similar allometric growth patterns. Digestive tract height showed negative allometric growth (*b* = −0.56) from hatching to 6.26 mm TL (2–3 DAH), followed by a sharp change to positive allometric growth (*b* = 6.62) until 10.01 mm TL (15–30 DAH). Digestive tract length showed negative allometric growth (*b* = 0.63) from hatching to 6.78 mm TL (6–7 DAH, before first feeding), and then positive allometric growth (*b* = 1.44) until 10.01 mm (15–30 DAH). This change in positive allometric growth in these digestive tract parts may be advantageous in terms of consuming more food at first feeding. Indeed, the average area of intestine decreased at 6 DAH (0.103 ± 0.015 mm^2^) compared to 5 DAH (0.122 ± 0.019 mm^2^), but increased sharply from 7 DAH (0.127 ± 0.007 mm^2^ at first feeding to 30 DAH (1.022 ± 0.189 mm^2^). Thereafter, digestive tract length and height changed from 10.01 mm TL (15–30 DAH) to close to isometric growth (*b* = 0.93, *b* = 0.95, respectively). A change to isometry in allometric growth patterns of teleost species during the early development stages has been interpreted as completion of the development of some of the primary physiological systems with the formation and maturation of several sensory and digestive organs [25,26,53]. Indeed, Shin et al. [64] reported that the histological differentiation of digestive organs of eel larvae was mostly completed by 15 DAH, and Shin et al. [65] found that digestive enzyme activity of eel larvae increased rapidly until 30 DAH. In other words, eel larvae show rapid development of the function and differentiation of the digestive system before 30 DAH, these rapid changes in the digestive system may be related to critical period I, II that occur at the same time. Hsu et al. [20] reported that the slurry-type diet is difficult to digest and absorb in the eel larval intestine. Therefore, eel larvae should be necessary to develop the optimal diets suitable for the ontogeny of digestive ability before 30 DAH.

## 5. Conclusions

This study was performed to determine the changes in morphology and growth pattern during eel larval stages. When cultured eel larvae were divided according to the morphological criteria of wild eel larvae, the sizes of TL that divided the stages were similar between cultured and wild eel larvae. However, cultured eel larvae differed from wild eel larvae in terms of growth rate and the number of preanal myomeres. These differences suggest that further advances are still needed in cultured eel seed rearing techniques. In particular, as eel larvae, which rarely have mixed feeding periods, do not feed immediately after mouth opening, further research is required to determine the optimal first feeding time. In addition, the lower jaw abnormality of eel larvae was assumed to be caused by the slurry-type diet supplied at the bottom of the tank, and a new diet should be developed for the leptocephalus stage (wherein the lower jaw is longer than the upper jaw). Meanwhile, the relative growth pattern of the body parts changed only before 30 DAH, and mass mortality appeared at this time. In particular, the allometric growth of digestive tract parts fluctuated rapidly during this period, consistent with histological and physiological events during the ontogeny of eel larvae. Therefore, to improve the growth and survival rate of cultured eel larvae, studies should focus on developing an optimal feeding and rearing protocol from the first feeding to 30 DAH. This study provided baseline information for understanding the early life history and improving optimal rearing protocols of freshwater eels.

## Figures and Tables

**Table 1:** Compositions of the slurry-type diets.

Ingredient	Diet
Shark egg ^1^ (g)	50
Fish soluble protein ^2^ (g)	3
Soybean peptide ^3^ (g)	3
Krill extract ^4^ (g)	6
Vitamin mix ^5^ (g)	0.3
Proximate composition (mean ± SD, *n* = 5)	
Moisture (%)	68.01 ± 0.39
Crude protein (%)	17.91 ± 0.15
Crude lipid (%)	10.42 ± 0.21
Crude ash (%)	0.73 ± 0.03

^1^ Eggs of spiny dogfish (*Squalus acanthias*). ^2^ Sopropeche. France. ^3^ Shandong Yuxin Biotechnology. China. ^4^ Krill (5 kg) was homogenized with distilled water (4 L) and incubated with a digestive enzyme mixture at 50 °C for 6 h, and then, the mixture was filtered through a nylon plankton net (mesh opening, 100 µm), lyophilized at −80 °C, and stored at −50 °C until diet preparation. ^5^ Vitamin mixture (per 1 kg dry matter): vitamin A, 5,000,000 IU; D3, 1,000,000 IU; E, 37,500 mg; K3, 2500 mg; C, 6400 mg; B1, 5000 mg; B2, 10,000 mg; B6, 5000 mg; B12, 25 mg; H, 50 mg; folic acid, 2500 mg; inositol, 75,000 mg; nicotinic acid, 37,500 mg; Ca-pantothenate, 17,500 mg. Diet viscosity was adjusted using sea water.

## Data Availability

Not applicable.
